# Ways to Assess and Regulate the Performance of a Bi-Mechanism-Induced Borneol-Based In Situ Forming Matrix

**DOI:** 10.3390/pharmaceutics15082053

**Published:** 2023-07-31

**Authors:** Nutdanai Lertsuphotvanit, Jitnapa Sirirak, Poomipat Tamdee, Sarun Tuntarawongsa, Thawatchai Phaechamud, Takron Chantadee

**Affiliations:** 1 Program of Pharmaceutical Technology, Faculty of Pharmacy, Silpakorn University, Nakhon Pathom 73000, Thailand; 2 Department of Chemistry, Faculty of Science, Silpakorn University, Nakhon Pathom 73000, Thailand; sirirak_j@silpakorn.edu (J.S.); tamdee_p@silpakorn.edu (P.T.); 3 Pharmaceutical Intellectual Center “Prachote Plengwittaya”, Faculty of Pharmacy, Silpakorn University, Nakhon Pathom 73000, Thailand; tuntarawongsa_s@su.ac.th; 4 Natural Bioactive and Material for Health Promotion and Drug Delivery System Group (NBM), Faculty of Pharmacy, Silpakorn University, Nakhon Pathom 73000, Thailand; 5 Department of Industrial Pharmacy, Faculty of Pharmacy, Silpakorn University, Nakhon Pathom 73000, Thailand; 6 Center of Excellent in Pharmaceutical Nanotechnology, Chiang Mai University, Chiang Mai 50200, Thailand; 7 Department of Pharmaceutical Sciences, Faculty of Pharmacy, Chiang Mai University, Chiang Mai 50200, Thailand

**Keywords:** borneol, triacetin, solvent exchange, in situ forming, molecular dynamics simulation

## Abstract

As an alternative to the traditional polymeric-based system, it is now possible to use an in situ forming system that is based on small molecules. Borneol was used as matrix formation in this study. While triacetin was incorporated into the formulation for prolonging the drug release. The objective of this study is to understand the initial period of the solvent exchange mechanism at the molecular level, which would provide a basis for explaining the matrix formation and drug release phenomena. The evaluation of basic physical properties, matrix formation, in vitro drug release, and molecular dynamics (MD) simulation of borneol-based in situ forming matrixes (ISM) was conducted in this study. The proportion of triacetin was found to determine the increase in density and viscosity. The density value was found to be related to viscosity which could be used for the purpose of prediction. Slow self-assembly of ISM upon the addition of triacetin was associated with higher viscosity and lower surface tension. This phenomenon enabled the regulation of solvent exchange and led to sustaining the drug release. In MD simulation using AMBER Tools, the free movement of the drug and the rapid approach to equilibrium of both solvent and water molecule in a solvent exchange mechanism in borneol-free ISM was observed, supporting that sustained release would not occur. Water infiltration was slowed down and NMP movement was restricted by the addition of borneol and triacetin. In addition, the increased proportion of triacetin promoted the diminished down of all substances’ movement because of the viscosity. The diffusion constant of relevant molecules decreased with the addition of borneol and/or triacetin. Although the addition of triacetin tended to slow down the solvent exchange and molecular movement from computation modelling results, it may not guarantee to imply the best drug release control. The Low triacetin-incorporated (5%) borneol-based ISM showed the highest ability to sustain the drug release due to its self-assembly and has proper solvent exchange. MD simulation addressed the details of the mechanism at the beginning of the process. Therefore, both MD and classical methods contribute to a clearer understanding of solvent exchange from the molecular to macroscopic level and from the first nanosecond of the formulation contact with water to the 10-day of drug release. These would be beneficial for subsequent research and development efforts in small molecule-based in situ forming systems.

## 1. Introduction

Currently, the utilization of a small molecule-based in situ forming system has recently emerged as an alternative to the conventional polymeric-based system. However, due to a dearth of comprehension and limited knowledge, managing the behavior and performance of this system proves to be challenging. Typically, polymers have been used as matrix former in ISM systems. However, problems still exist in polymer-based ISMs such as the high viscosity of prepared ISM (solution state), burst release effect, and removal of formed matrix [1]. The difficulty in delivering preparations through the needle caused by high viscosity is a problem for drug administration. Furthermore, such viscosity and slow matrix formation result in an out-of-control phenomenon during the early phase of drug release. Therefore, non-polymers ISM is interesting to investigate. Prior research has indicated that the performance of the aforementioned factors can be influenced by various solvents, structural forming agents, and concentrations of matrix-forming agents [2,3]. The objective of this study is to understand the initial period of the solvent exchange mechanism at the molecular level, which would provide a basis for explaining the matrix formation and drug release phenomena. Moreover, this study was also to determine a simplified method for assessing the in situ forming capabilities, which could be applied in laboratory or industrial settings, where the expenses associated with experimentation are significant. This would facilitate further research and development efforts on small molecule-based in situ forming systems.

**Figure 1:**
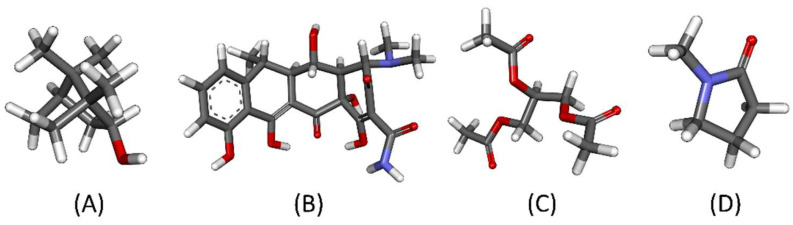
3-D structure of borneol (**A**); doxycycline (**B**); triacetin (**C**) and NMP (**D**). Gray, white, red and blue sticks represent C, H, O, and N atom, respectively.

An in situ forming system is a drug delivery system that begins as a liquid dosage form and then transforms into solid-like forms at the target site, which can sustain drug release and maintain drug levels at the target site. Given that it is a liquid dosage form, this system has been designed to administer wherever it suits to be injected, with less pain than other solid implants [4,5]. A solvent exchange-induced in situ forming matrix (ISM) is an injectable drug delivery system that dissolves its active ingredients in biocompatible solvents. After being administrated into an aqueous environment, the solution transforms to a matrix-like state due to the influx and out flux migration of anti-solvent (aqueous fluid) and solvent, respectively. The entrapped drugs are liberated from the precipitated matrix over time [6]. These systems can deliver the drug locally to improve its concentration around the injection site and reduce systemic toxicity [7,8]. Solvent-exchange-ISM development begins with a polymer-based system and progresses to various hydrophobic materials. Several hydrophobic materials, such as phospholipid, cholesterol, and fatty acid, have recently been used as structure forming components [9,10,11,12]. These materials promote a different unique ISM character than traditional ISM. Borneol, a highly lipid-soluble (Log K_o/w_ = 2.85) bicyclic monoterpene alcohol, is typically found in several natural products such as nutmeg, ginger, thyme, and citrus peel oils [13,14]. It has been used as an active ingredient for the relief of the following conditions: local itching, bronchitis, minor aches and pains in muscles and joints. It also has antibacterial and anti-nociceptive properties [13]. In terms of pharmaceutical drug carriers, it was incorporated into solid lipid nanoparticles (SLNs) using a modified microemulsion method. Borneol-modified SLNs are an effective method of delivering drugs to the brain [15]. In human volunteers and mice/mice, it has been reported that borneol has a low toxic potential for causing skin irritation [14]. Thymocytes were grown in an environment with borneol at concentrations of 0.5 and 5 g/mL. This clearly showed that borneol did not make immune system cells toxic [16]. Moreover, borneol exhibits anti-fibrotic activity, possibly through the inhibition of fibroblast mitosis, production of collagen, and production of TIMP-1 [17]; thus, this compound is regarded as safe for the treatment of oral submucous fibrosis. Although borneol is not classified as a polymeric as other matrix-forming agents due to its structure shown in Figure 1A, borneol has possibly been used as a matrix-forming agent owing to these properties as mentioned. Given its long-term safety and apparent low aqueous solubility, borneol may serve as a matrix-forming component of anti-solvent based-ISMs.

In situ forming systems often have problems with burst release. To make this system better, researchers have tried to control the system’s behavior in different ways. Several liquids that could change the flow of the solvent exchange rate have been looked at. Clove oil was found to restrict the rate of solvent exchange, which changed the way the material formed and made it less likely to burst [8]. The concept of mixed solvent was also a way to modify the migration ability of the solvent, which resulted in several different behaviors during the formation process [18]. Triacetin, a triester of glycerol and acetylating agents (acetic acid and acetic anhydride) [19], is safe as it is reported on the FDA’s GRAS list and has not been found to be toxic in short-term inhalation or parenteral studies [20]. Since the LD50 for triacetin is more than 2 mL/kg, it could be used safely in injectable implant systems [21]. It was used as a plasticizer [22] and co-solvent in order to minimize a burst release and extend the drug release of PLGA-in situ forming systems [23]. For hydrophilic polymer depots, using triacetin as a cosolvent with *N*-methyl-2-pyrrolidone (NMP) or dimethyl sulphoxide resulted in less burst release effect [24]. Therefore, triacetin was chosen to investigate the way to control non-polymer-based ISM behavior.

According to the few reports on this ISM subgroup, it is unclear why solvent-exchange has been considered as an activating key rather than a main role mechanism (crystallization) [25]. Prior research focused on the macro and microscopic behavior of formation, the topography of a formed matrix, the diffusion of solvent and aqueous phase, and the various physical and chemical properties involved in the solvent exchange mechanism [3,9,10,12,26]. Nevertheless, the underlying in situ formation mechanism of this subgroup is still unclear. The current study used molecular dynamics (MD) simulation to better understand the mechanism underlying this pharmaceutical dosage form and its macro/microscopic properties. Such an experiment of MD was used and discussed in conjunction with the multiple results including the matrix formation results observed at macro and microscopic levels [11,12]. Using computational analysis and MD simulation, it is possible to determine the fundamental process to understanding this movement.

Borneol was selected as the structure former of a doxycycline-loaded ISM in which borneol was dissolved in NMP and triacetin at different ratios. The structures of borneol, doxycycline, triacetin, and NMP are shown in Figure 1A–D. This investigation of the ISM formulation consists of the evaluation of basic physical properties of the formulation, matrix forming behavior, interfacial behavior, in vitro drug release, and the study of computer dynamics modelling at the molecular level. The goal of this investigation was to acquire more about the early stages of the in situ formation process, the molecular flow of components, and the interactions between the drug and the borneol matrix, all of which affect how well this system works, such as the level of burst release, its ability to form itself, and its ability to keep releasing drugs.

## 2. Materials and Methods

### 2.1. Materials

The procurement of borneol was carried out at the Chareorsuk-osop herbal shop located in Nakhon Pathom, Thailand. The experiment involved the preparation of sodium hydroxide (lot no. AF310204, Ajax Finechem, NSW, Australia) and potassium dihydrogen orthophosphate (lot no. E23W60, Ajax Finechem, NSW, Australia) in phosphate buffered solution (PBS) having a pH of 6.8. Agarose (Lot No. H7014714, Vivantis, Selangor Darul Ehsan, Malaysia) was used to determine the gel formation behavior. The model drug utilized in this study was doxycycline hyclate (lot no. 20071121, Huashu Pharmaceutical Corporation, Shijiazhuang, China). The regulating agent utilized in the study was triacetin, sourced from Acros Organics in Nidderau, Germany and identified by lot number A0375797. The substance referred to as NMP (lot no. 144560-118, QReC, Auckland, New Zealand) was utilized as a solvent.

### 2.2. Preparation of Formulation

The 5%*w*/*w* doxycycline hyclate and 40%*w*/*w* borneol with various triacetin concentrations were prepared by continuously mixing for 3 h with a magnetic stirrer (50RPM) at room temperature (25 ± 1 °C) until clear solutions were obtained, using NMP as a solvent. The composition of formulations is shown in Table 1.

### 2.3. Evaluations of Physical Properties of Liquid State ISM: Density, Apparent Viscosity, and Surface Tension

The density of triacetin, NMP, and preparations was measured at room temperature using a density meter (Densito 30PX, Mettler Toledo Ltd., Vernon Hills, IL, USA) (*n* = 3). The Brookfield rheometer (Brookfield DV-III Ultra, Brookfield Engineering Laboratories Inc., Middleboro, MA, USA) was used to determine the viscosity of triacetin, NMP, and the preparations. The speed of the spindle was set at 150 rpm (*n* = 3). The surface tension of triacetin, NMP, and preparations was determined by observing the transformation that took place in the shape of a pendant drop of a formulation (approximately 50–60 µg) while it was suspended in air following injection with a goniometer (FTA 1000, First Ten Angstroms, Newark, CA, USA) set to a pump out rate of 1.9 µL/s (*n* = 3).

### 2.4. Matrix Forming Behavior of Borneol-ISM

The transformation behavior after the liquid formulation exposure of the aqueous medium into the matrix phase was checked. The 1 mL of preparations was injected through the 27-gauge stainless needle into 5 mL of PBS pH 6.8 in a glass test tube. The matrix formation behavior was observed and photographed at various times (0, 5, 10, 15, and 20 min).

### 2.5. Interfacial Behavior of Borneol-ISM 

The interaction at the interface between the formulations and the simulating gum (formed agarose with phosphate buffer) is studied. The simulating biological fluid, phosphate buffer solution pH 6.8 (PBS), was prepared. The pH of 6.8 buffer was chosen because it corresponded to the pH of the crevicular fluid in the periodontal pocket and saliva in the mouth. The 300 μL of simulating fluid was dropped on the glass slide. After that, the sample of 100 μL formulation was then dropped onto the glass slide, and in contact with the previous drop of PBS. The morphological changes were caused by phase inversion at the interface and photographed under a stereomicroscope (SZX10, Olympus Corp., Tokyo, Japan) (magnification of 8×) at various time intervals.

### 2.6. In Vitro Drug Release

The modulation of drug release with the borneol matrix was evaluated. The doxycycline hyclate release from ISM and a control (doxycycline hyclate in NMP) were undertaken via the cup method. A cylindrical-shaped porcelain cup (1 cm in diameter and 1.2 cm high) containing 0.4 g of the formulation was immersed in 50 mL of PBS (pH 6.8) at 37 °C, and the rotation shaker speed was maintained at 50 rpm using an incubator (Model NB-205, N-Biotek, Gyeonggi-do, Republic of Korea). The release medium was sampled (5 mL) and replaced with fresh PBS (5 mL). The collected medium sample was diluted with PBS to achieve a suitable range of absorbance (0.03 to 1.26 AU). The absorbance was then calculated using the doxycycline standard curve. A UV-Visible spectrophotometer (Cary 60 UV-Vis, Model G6860A, Agilent, Selangor, Malaysia) was used to measure the concentration of drug release at 280 nm and calculate the concentration of drug and amount of drug. The amount of drug released at each time point was summarized and calculated as the cumulative drug release. Each sample was repeated 6 times (*n* = 6) and averaged as the cumulative mean drug release at each time point with S.D. value. Finally, all data were plotted as % of drug release versus time. The standard curve of doxycycline in PBS pH 6.8 was performed by preparing standard concentrations of doxycycline at concentrations of 1, 10, 20, 30, 40, and 50 µg/mL (with absorbance in the range of 0.03 to 1.26 AU) using the same model of UV-Visible spectrophotometer at 280 nm. The release kinetics were inquired using the DD solver add-in program for Microsoft Excel (Redmond, WA, USA). The 10-80% cumulative percentage of drug release profiles was fitted with the following model: zero-order, first-order, Higuchi’s, and Krosmeyer-Peppas equations. The coefficient of determination (r^2^) was determined to indicate the level of fitness. The value of “*n*” in Krosmeyer-Peppas in dictating the mechanism of drug release. For a non-swelling system, it is Fickian diffusion if the *n* is close to 0.45, and anomalous diffusion if *n* value exceeds 0.45 but less than 1. Fickian diffusion indicates the less effect of structure relaxation than that of drug diffusion. Anomalous diffusion (non-Fickian) occurs when active diffusion is close to structure relaxation [27,28,29,30].

### 2.7. Computer Dynamics Modelling of Mechanistic Phase Inversion

#### 2.7.1. MD Simulation of ISM Formulation

To understand and support the phase inversion and molecular movement of each ISM formulation in an aqueous environment, computer dynamics modeling was performed. The initial coordinate of doxycycline, borneol, NMP, and triacetin molecules were taken from the Pubchem and Cambridge Crystallographic Data Centre (CCDC) database. The antechamber and parmchk2 in the AMBER20 software package [31] were then employed to prepare the topology files and frcmod for all substances. The simulation models of formulations (F1–F4) containing doxycycline, borneol, NMP, and triacetin molecules were designed based on the molar ratio. After that, the designed molecules were arranged and built as the 3D box model. Box design details which showed the molecular amount of each substance, mole ratio, and box size were displayed in Table 2A. Afterward, GaussView06 and UCSF Chimera [32] were utilized to rearrange the molecules in each system, and the models were built using the LEAP module in AmberTools. The energy minimization and 310 K temperature heating were performed using the sander module followed the MD simulation at 310 K with constant pressure for 200 ns using Amber20.

#### 2.7.2. MD Simulation of Interfacial Behavior of ISM with Water

A preliminary study about water sensitivity for matrix formation found that the minimum water content used to indue ISM solution and transform to the matrix was 20%*w*/*w* of the total substance. Thus, the ratio of formulation and water was 4:1 calculated based on weight. This ratio was used to design and arrange molecules in a 3D box model of each formulation with water for observing the phenomena of phase inversion at the interface when the formulation had contact with water. The amount of each substance and their ratio were calculated and shown in Table 2B. The geometry of the last snapshot of each simulated drug–borneol–NMP/triacetin systems above were collected and placed next to the box of TIP3P water. Additionally, the models were built using the LEAP module in AmberTools. Afterward, energy minimization was conducted followed by heating up to 310 K using a sander module and MD simulation at 310 K with constant pressure for 200 ns using Amber20. Moreover, Visual Molecular Dynamics (VMD) [33] was utilized to analyze and image all simulations.

#### 2.7.3. Simulation Analysis

The hydrogen bond (H-bond) occupancy was calculated using visual molecular dynamics (VMD) software [33]. The criteria for H-bond formation are that the distance between the acceptor and donor atoms is less than 3.5 Å and the angle made by the acceptor, donor and hydrogen atoms is less than 60° [34]. The root-mean-squared deviation (RMSD) of matrix, borneol, NMP and doxycycline was obtained using the cpptraj module [31]. The ptraj module with the simulation time-evolved trajectory was used to calculate the diffusion constants which refer to the distance migrated from its initial position. The ability to migrate outflux/influx of doxycycline, borneol, NMP, triacetin, and water was pointed out by the mentioned constant [31].

### 2.8. Statistical Analysis

The data was presented as mean and standard deviation (mean ± S.D.). For statistical analysis, SPSS for Windows was employed. The measurement’s statistical significance was examined and considered at *p* < 0.05 using one-way analysis of variance (ANOVA) followed by an LSD post-hoc test.

## 3. Results and Discussion

### 3.1. Density

Given that density is a fundamental property that can be easily measured and has the potential to serve as a predictor for other properties of small molecule-based in situ forming systems [3,7], such as injectability, viscosity, and matrix formation. Moreover, the density of the preparations was the crucial parameter for surface tension measurement. In addition, the density greater than the density of water is a confirmation that the formulation will settle down in the periodontal pocket throughout the administration. Such properties give the formulation a chance to adhere firmly in the periodontal pocket, resulting in effective treatment [3]. The density of all formulations was measured and reported in Table 3. The density of all formulations was greater than that of water (1.00 g/cm^3^); thus, they are possible to firmly adhere in the pocket. The inclusion of borneol in the system resulted in a lower density compared to the solution comprising only the drug in NMP (control group). Typically, the density of ISM (solution state) is the sum of the densities of each component based on their respective mass fractions [35]. The substitution of NMP with borneol addition was responsible for the reduced density of F1. Borneol was utilized as a matrix formation agent resulting in a comparable density to that of other small molecule-based in situ forming systems such as fatty acids-based in situ forming systems. The study demonstrated that the density of fatty acids-based in situ forming system with a concentration of 35%*w*/*w* varied between 1.0109 and 1.0240 g/cm^3^, which depended on the length of the aliphatic chain. Specifically, the fatty acids with shorter aliphatic chains exhibited higher densities in comparison to those with longer chains [7]. Moreover, This study conducted on the borneol-based ISM revealed that the density values varied with the amount of triacetin used. Namely, an increasing trend in density was observed with an increasing ratio of triacetin (*p* < 0.05), which was attributed to the inherent density of triacetin and the reduced proportion of NMP in the formulations.

Furthermore, the correlation between density and the values of viscosity and surface tension was observed, with the exclusion of F1 which was non-ISM (control). This finding corroborates the prior research indicating that density may serve as a viable means of approximating the basic characteristics of any in situ forming system using small molecules as structural forming agents. Nevertheless, it presented a constraint. As mentioned before, density is a sum of density values for each of the components. Hence, in order to derive estimations for additional properties through the utilization of density, the variable factor should be limited, only one variable is allowed to be altered. It should be compared within the same system of preparation. The non-ISM solution (without matrix former) or ISM with a different matrix former should not be compared. For example, the inclusion of F1 in determining the relationship between density and other properties was found to be inadequate due to its inability to establish a correlation as it did not have an in situ forming system. Particularly, it had two variable factors: borneol concentration and triacetin concentration.

### 3.2. Viscosity

The viscosity of all ISM formulations and relative solvents is shown in Table 3. Typically, NMP is a low viscous organic solvent; thus, ISM formulation containing NMP exhibited low viscosity. The viscosity of borneol-based ISM was rather higher than that of borneol-free formulation due to a strong hydrophilic interaction between borneol and solvent as well as the reduction of the solvent in the formulation [3]. While Triacetin is a hydrophobic triglyceride ester with a viscosity of 25 cPs. Consequently, the addition of triacetin to the prepared ISM usually resulted in a slight increase in viscosity. The augmentation of the triacetin proportion resulted in a substantial rise in viscosity (*p* < 0.05), suggesting the potential to regulate diverse ISM phenomena, including solvent-exchange, self-assembly, and drug release management. The ease of solvent and anti-solvent migration was influenced by viscosity. The formulation with high viscosity exhibited a slow solvent migration, leading to a decelerated self-assembly phenomenon [11,12]. In the presented borneol, the viscosity of the solution was increased [3]. The elevated proportion of triacetin led to an increase in the viscosity of the solution, which was related to the inherent viscosity of triacetin. According to the H-bond interaction of each molecule, increase in viscosity was observed [25]. Triacetin, which contains multiple functional groups capable of forming H-bonds, exhibited intermolecular interactions due to the high density of triacetin-loaded preparations.

Although the system viscosity was high by the increasing of triacetin, all preparations were deemed suitable for injection because their viscosity was less than 20 cPs [11]. The developed system presented with a lower viscosity than the hydrogel-based injectable in situ forming system [36]. Furthermore, the viscosity was lower than that of other injectable thermo-responsive in situ forming gel systems, such as carbopol-poloxamer gels, which have viscosities in the range of 19,000–36,000 cPs at 4 °C [37,38]. The low viscous of the borneol solution was due to the fact that it was low density solution and the borneol has a small molecular size with simple structure resulting in less molecular interactions. Rheological character was an important parameter for the ISM as it indicates ease of drug administration through a syringe [1]. The exponential constant of F1, F2, F3, and F4 was 0.96, 0.98, 0.98, and 0.97, respectively. They were close to 1.00 indicating the viscosity of the formulation remains constant even with increasing shear rate. These indicated Newtonian flow behavior, which the shear rate was directly proportional to the shear stress. This flow behavior was suitable for ISM because the formulation requires force to be expelled from the syringe via a small needle. Namely, the force used for injection does not affect the viscosity change and leads to in ease of administration [39]. Moreover, it was reported that the temperature did not change the flow behavior of polymer-based ISM but decreased shear stress in ranges of 25–37 °C [40].

Hence, the fluid state of the developed systems, which was less viscous which refers to a high injectability, provided the potential to minimize the painful application that can occur during injection through the needle and the ease of use.

### 3.3. Surface Tension

The direct determination of interfacial tension between preparations and aqueous fluid was not achievable due to the immediate occurrence of phase transition at the surface of the system droplet. Consequently, the focus of the investigation was on surface tension instead of interfacial tension. The surface tension of all ISM formulation and relative solvents was displayed in Table 3. According to the steric hindrance effect of borneol, the surface tension of the borneol-based ISM was less than that of the borneol-free formulation. Moreover, borneol at 40% also interferes with the interactions between each solvent molecules results in a lower surface tension [2,3]. With the addition of triacetin, the surface tension gradually decreased while the viscosity gradually increased. The observed outcomes imply that triacetin also disrupted the interactions among the NMP-NMP molecules. The reduction in surface tension typically implies the absence of barriers for the solvent exchange, thereby enabling alternative approaches to regulate the exchange dynamics. However, there was an additional aspect to consider. In the case that the structural agent was a small molecule with the ability to crystallize, the reduction in surface tension would indicate a lowered interfacial energy within the system, thereby leading to slow self-assembly behaviors. Therefore, it is necessary to consider multiple parameters and not just a singular one. The evaluation of formation behavior, interfacial interaction, and drug release was conducted for the purpose of demonstration.

### 3.4. Self-Formation Ability of ISMs

**Figure 2:**
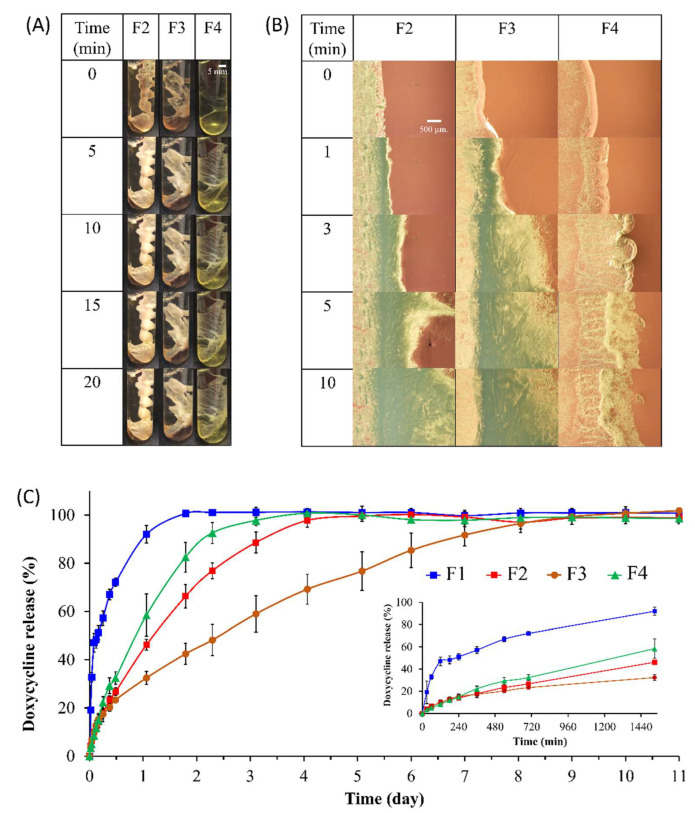
(**A**) Matrix formation of borneol-based ISMs in PBS pH 6.8 at macroscopic level; (**B**) interfacial network formation of borneol-based ISMs under a stereomicroscope (magnification of 8×); (**C**) doxycycline release profile of ISM formulation and control (F1) in PBS pH 6.8 (*n* = 6).

The self-formation behavior, which is the essential performance of the in situ forming system, was evaluated. The gel-like state of the prepared formulations upon injection into the PBS pH 6.8 buffer at several time points is shown in Figure 2A where the opaque referred to the completeness of formation [8]. Usually, the prepared borneol-based ISMs were homogeneous yellowish with clear solutions. As it was contacted with PBS pH 6.8, the yellowish ISM of the solution state was transformed into a white color indicating a gel-like white matrix formation. The white matrix was occurred from the white borneol crystals which precipitated through a phase separation process. However, the formulations that slow transformation rate or incomplete matrix formation may appear the yellowish color from the doxycycline. The gelling time of F2, F3, and F4 after transforming from the transparent ISM solution into the opaque white matrix was 5 min, 5 min, and >20 min, respectively. The borneol-based ISMs formulations had compact surfaces, whereas the increased triacetin formulations contained a soft surface and yellowish color indicating that triacetin could affect the formation behavior. Triacetin significantly delayed the completeness of ISMs matrix formation. The high triacetin-loaded ISM (F4) showed a slow matrix formation and could not transform to the solid-like matrix within 20 min. Whereas triacetin-free ISM (F2) and low triacetin-loaded ISM (F3) could change to the solid opaque matrix within 5 min, which tended to be possible to control the drug release. These are relevant to the viscosity value of the preparation, where the high viscosity resulted in the difficult migration of environment fluid inward and system fluid outward. The high triacetin ratio preparation (F4) showed limited formation due to the following reasons: Hydrophobic solvents, such as triacetin, have been observed to facilitate a slow phase inversion process [41]. It was reported that solvents with a water solubility below 7%*w*/*w* have been demonstrated to cause a decrease in water uptake, leading to a slower drug release [42]. The gel formation process was observed to be slowed down due to the hydrophobic nature of the triacetin (compared to NMP), which hindered water penetration. Moreover, at high levels of triacetin concentration, the solvent exchange was slower than the interfacial network formation. Subsequently, the liquid migration was blocked as the interfacial network of the initiated borneol matrix became denser with time with less porosity and high tortuosity. It was reported that the compact gelation structure resulted in a decrease in the coefficient of water diffusion [43].

Given that fluid migration is prohibited, the solvent exchange mechanism is stopped. The self-formation of small molecule-based in situ forming systems stays in progress. The other mechanism, the continuous growth of crystals, occurred as a second mechanism. This rate of secondary self-formation was dependent on the ability of borneol crystallization in a given triacetin ratio at a given time. Theoretically, any unmixable object has an energy at its boundaries that enables nucleation and subsequently crystallization through a thermodynamic driving force [44]. The thermodynamic stability of a system can be disrupted by a variety of factors, including the introduction of new interfacial energy from sources such as the initial crystal, aqueous surface, and bubble surface, as well as supersaturation resulting from solvent reduction. As the thermodynamics are disturbed, the system will stabilize itself by activating crystal formation [45]. Therefore, surface tension which is related to the activating key of crystallization in thermodynamics term, surface free energy, was evaluated. Namely, the high surface tension results in ease of nucleation. As mentioned above, not only viscosity might affect the formation behavior, but also surface tension. The high portion of triacetin such as F4 showed a low surface tension which resulted in less thermodynamics driven energy. Subsequently, the nucleation was slower than that of less triacetin preparation including F2 and F3.

Not only how fast nucleation and crystallization need to be concerned, but also the number of nucleation origins at initial the time point. The high number of nucleation origin resulting in less porosity and high tortuosity. While the less nucleation origin showed less tortuosity [25]. Finally, as the formation behavior changes, the release of drugs might be affected [12]. However, it should be noted that the suitable formation speed depends on the purpose: burst control release, immediate release, sustaining release, etc. These statistics only provide management options. The suitable preparation properties are neither a fast formation nor a slow formation. For example, too fast formation may result in non-fitting with the target shape, while too slow formation may result in leakage from the target site. Hence, it is possible to predict the formation behavior for future research or industrial product development by utilizing essential physical properties such as viscosity and surface tension. The use of density is likewise relevant as it facilitates the prediction of both viscosity and surface tension.

### 3.5. Interfacial Network Formation of ISMs

To verify the impact of the interfacial network on the influx/outflux of relevant fluids and the formation rate. The boundary between preparations and PBS pH 6.8 (a representation of biological aqueous fluid) was tracked to achieve interfacial network formation as shown in Figure 2B. The aqueous phase was placed on the right side, while the preparations were located on the left. 

The absence of triacetin facilitated rapid networking at the interfacial boundary. As can be seen, the F2 formulation (triacetin-free ISM) had more formation at the third minute than the F3 formulation.

Solvent exchange was hampered when the self-networking at the interfacial boundary was occurred. However, the F2 formulation continued to undergo self-formation over time. This could be due to several reasons, including the presence of enough porous to allow the related fluid to exchange or the occurrence of a secondary mechanism such as crystal growth as mentioned. In practice, both of the aforementioned factors occurred concurrently. Particularly, the exchange cannot be completely blocked at the given time otherwise, the drug would be unable to be released from the system. Due to thermodynamic instability, borneol is precipitated. It is probable that both mechanisms were concomitant, albeit one mechanism may have exhibited greater salience at a particular time. Nonetheless, both mechanisms had an end-point. Regarding the first mechanism, solvent exchange, the solvent either underwent complete diffusion out of the system or encountered significant hindrance in its diffusion, leading to a state of negligible diffusivity [46]. The second mechanism, crystal growth, terminated when the thermodynamics became stable [44,45]. These findings suggested that borneol-based ISMs had two mechanisms for self-formation.

The highest triacetin ratio preparation, F4, behaved very differently than the low triacetin formulation. The results were according to the previous discussion, the low surface tension formula (F4) showed less self-formation than the high surface tension formula (F2) due to the lower activating energy. The high viscosity of F4 was the other reason that resulted in less solvent exchange.

Relying solely on a standalone parameter for prediction can result in errors. Precisely, because the surface tension of F3 was lower than that of F2, the transformation of F2 should be faster. But the self-formation of F3 was greater than F2. This indicated that several parameters should be considered. The initial boundary network formation of F3 was faster than that of F2 because the viscosity of the solution and hydrophobicity of triacetin retarded the solvent exchange, resulting in a less dense network at a given time [41]. The less dense initial network refers to the higher porosity, which allowed the processing of solvent exchange. Namely, at the initial time, the F3 possessed two mechanisms that synergized with each other to generate the surface network. While the less porosity of F2, the second mechanism dominated the first mechanism because the solvent exchange was prohibited.

### 3.6. In Vitro Drug Release

The formation of a matrix is an essential prerequisite for achieving a sustaining release of drugs from an in situ formation system. According to Section 2.6, the standard curve of doxycycline was performed in the range of 1 to 50 µg/mL. The absorbance was linearly related to the concentration of doxycycline, with the equation of absorbance = 0.0248 × (concentration) + 0.002 and r^2^ = 0.9999). The drug release study was performed in which 5%*w*/*w* doxycycline hyclate in NMP (Control, F1) and ISMs preparations (F2, F3, and F4) are selected. The release profile of all formulations are shown in Figure 2C. The result indicated that the F1 exhibited characteristics of a drug release system that was non-sustained. Doxycycline hyclate in F1 was completely released within 2 h with a burst release at the initial phase of the drug, whereas F4, F2, and F3 presented a prolonged release up to 4, 5, and 10 h, respectively. The F3 showed none of the initial burst release as presented in Figure 2C. These improvements corresponded to the formation of borneol matrix and the triacetin loading. Namely, the drug slowly migrated through the borneol solid matrix structure since the drug was entrapped in the formed matrix. Borneol has been used as matrix former for control the drug release [3]. Currently, prolonging the release of drugs is a new challenge in the development of drug delivery systems. A possible approach for managing this issue is to diminish the hydrophilicity of ISM system by adding a hydrophilic substance [47]. Triacetin has been used as a hydrophobic solvent in polymer-based ISM to slow phase inversion, limiting solvent diffusion and retard the drug release [24,47]. However, the proper amount of triacetin added is an important consideration [24]. The different release profile from each matrix structure was due to the different interior structure, where the high tortuosity and less porosity provided the high ability to sustain the release of drugs. Because the high tortuosity and less porosity structure prolonged the migration of fluid, the drug diffusion with water was relied on this reason [3,25]. The release profile of F1, F2, and F4 was fitted well with the first order equation (r^2^ = 0.9831, 0.9889, and 0.9943 respectively). Interestingly, F3 release profile showed none of the initial burst release and was fitted with the Higuchi equation (r^2^ = 0.9969). The expedition of drug through the tortuosity of the triacetin added-hydrophobic borneol matrix, which accounted for the distance to the surface of the drug dissolution region, was claimed [48]. Namely, the actives diffusion out was slowed by the internal structure of borneol matrix. Moreover, the F3 release profile also fits well with the Krosmeyer-Peppas equation (r^2^ = 0.9974), where *n* = 0.51 indicating the anomalous diffusion (*n* = 0.45–0.89 for non-swelling system), where a mechanism of drug release is equally influenced by both of the diffusion and structure relaxation [48]. Although, Fickian diffusion, which structure relaxation shows less effect on drug release ability, often found in a matrix sustained drug delivery system [49]. The in situ forming system which required a structure formation process could result in a notably relaxation which influenced the release of drug than that of other sustaining drug delivery system.

Moreover, the hydrophobic fluid could retard the diffusion of the aqueous phase in which the drug diffusion was sustained, which corresponds to the report that clove oil could minimize the burst release of the drug [8]. The deceleration of movement was similarly noted when employing alternative hydrophobic matrix formers, including bleached shellac and cholesterol [9,50]. As previously stated, however, the integration of the overall factor is crucial. The utilization of an inappropriate proportion of triacetin generated opposite outcomes. An excessive triacetin ratio delayed matrix formation, leading to a highly porous surface network. This, in turn, facilitated the free-floating movement of the drug with fluid migration, resulting in a burst release. The deficient matrix formation of excessive triacetin preparation exhibited worthlessness in ability to sustain drug release and control burst release effect, comparison to the non-triacetin formula. The non-triacetin formula, F2, showed a burst release due to the fact that the matrix formation occurred rapidly with no prohibition effect. Namely, the only way to escape this rapidly forming matrix barrier of F2 was to move directly and massively; as a result, a large uncomplicated tunnel was formed, resulting in a high burst release and less ability to sustain drugs release.

A large passage might be another reason. It was reported that the rapid crystal formation of crystal-based ISM resulted in a large passage in the matrix. This was due to the smaller number of nucleation origin, the crystal growth from less number of nuclei resulted in large passage. The growth of each crystallization origin that was accompanied by vignettes led to a reduction in porosity and an increase in tortuosity [7]. F3, in contrast, which had triacetin, required more aqueous fluid influx to induce phase separation. Therefore, at the time point before the separation, a large aqueous-containing volume was obtained in this particular system. This high aqueous portion in the systems resulted in a high number of borneol nuclei, which coincided with the retarded crystal growth rate of each other, subsequently [47]. Due to the high number of nucleation origins and slow crystallization rate, the F3 interior formed a structure was less porosity and high tortuosity. Hence the sustaining drug release ability was achieved.

### 3.7. Computational Results of MD Simulation

**Figure 3:**
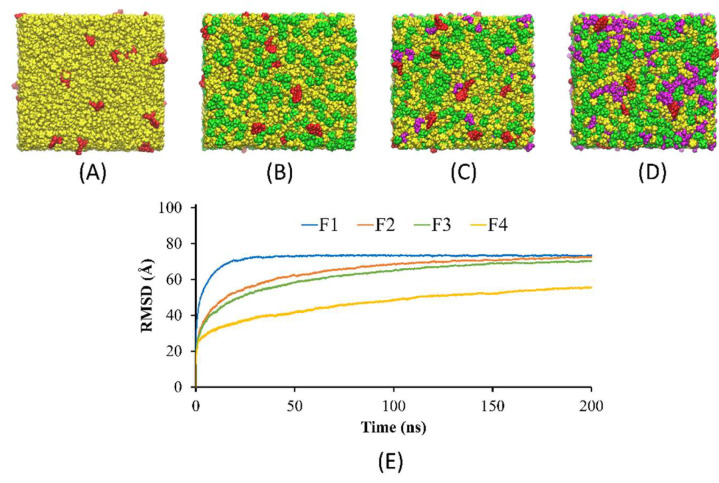
Molecular modeling of (**A**) F1, (**B**) F2, (**C**) F3, and (**D**) F4 after 200 ns running in MD simulation (red, green, violet, and yellow molecules represent doxycycline, borneol, triacetin, and NMP molecules, respectively), and (**E**) the RMSD of F1–F4.

The present study aimed to investigate the solvent exchange mechanism of the borneol-based formulations (F1–F4) containing doxycycline, borneol, triacetin, and NMP molecules at the very first initial time after contact with water. First, the models of F1-F4 were rearranged, simulated, and analyzed. As shown in Figure 3A–D, the components in the models mixed evenly within 200 ns of simulation time, indicating that all formulations exhibited homogenous formulations, which agreed with the experimental results showing that doxycycline hyclate dissolved and mixed well with other components in all formulations. Furthermore, Figure 3E demonstrated that F1 equilibrated after 30 ns and maintained a level of 73 Å, while F2–F4 reached equilibrium after 125 ns and remained constant at 70, 68, and 55 Å, respectively. It can be noticed that F2–F4 took a longer time to equilibrate than F1, which could be due to the interference of the borneol molecules in the system. Moreover, the RMSD of F4 at equilibrium was much lower than those of F1–F3 due to the higher amount of triacetin and also a higher viscosity of F4.

**Figure 4:**
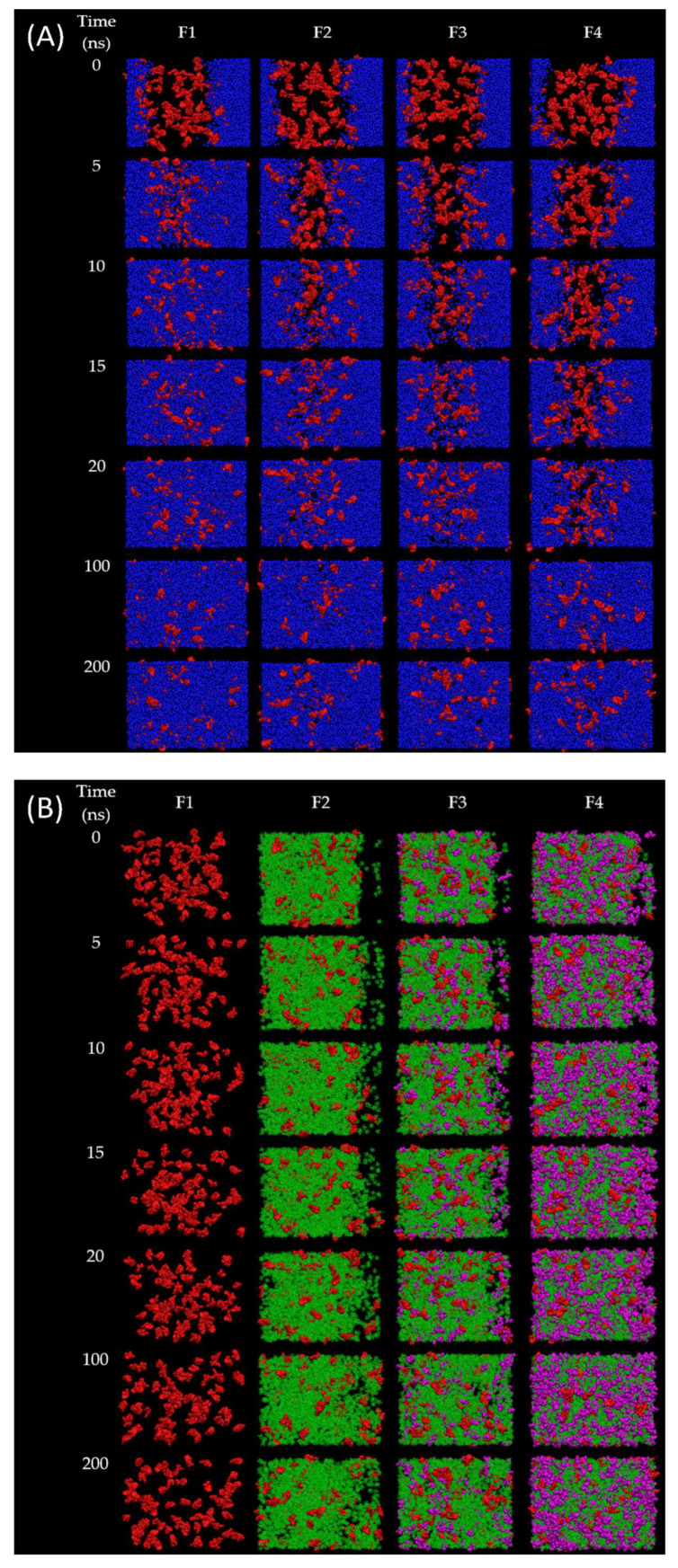

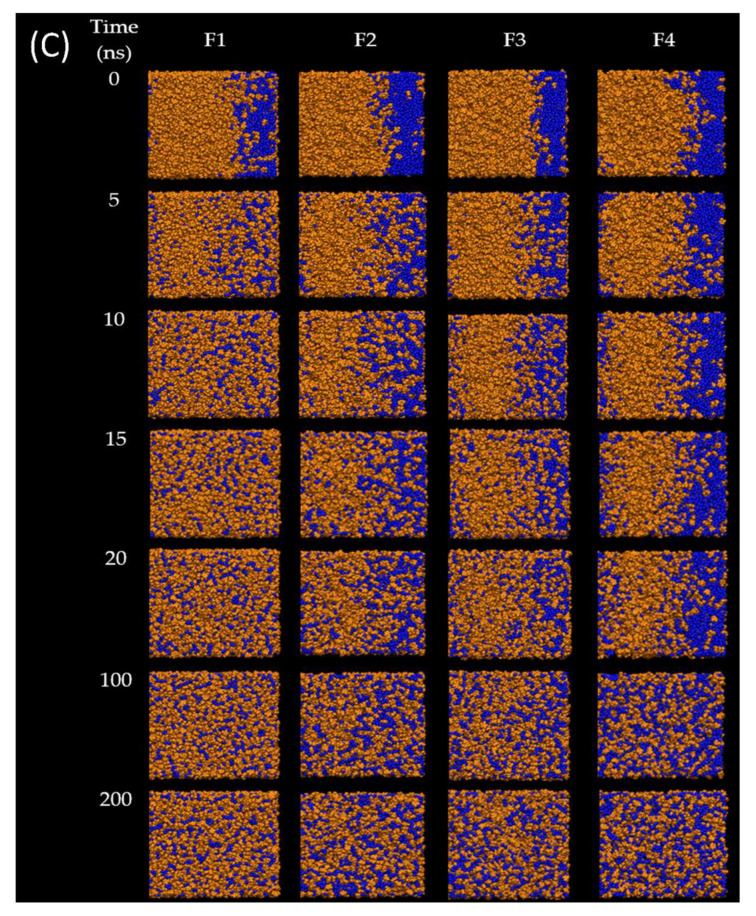
Molecular dynamic of solvent exchange mechanism focusses on: (**A**) doxycycline and water; (**B**) doxycycline, borneol, and triacetin; (**C**) NMP and water from MD simulation of different formulation box after contact with water box (red, green, violet, and orange molecules represent doxycycline, borneol, triacetin, and NMP molecules, respectively).

**Figure 5:**
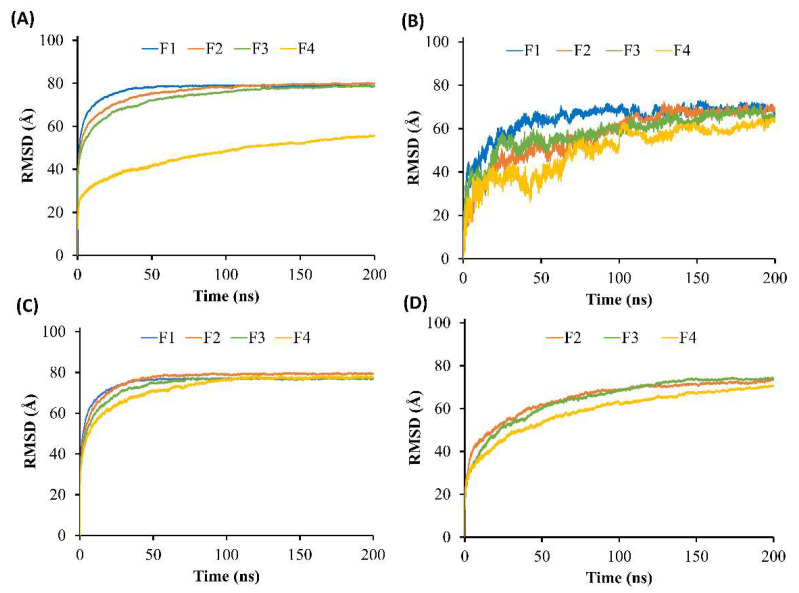
(**A**) The RMSD of all compartments of F1–F4, (**B**) RMSD of doxycycline conformation, (**C**) RMSD of NMP conformation, and (**D**) RMSD of borneol conformation relative to the starting point of the MD simulations box contact with water.

In order to observe the solvent exchanges of borneol-based formulations (F1–F4) upon the diffusion of water, the water-involved dynamic simulations of F1–F4 were performed for 200 ns. The snapshots during simulation time of 0, 5, 10, 15, 20, 100, and 200 ns of F1–F4 after contact with water were displayed in Figure 4, and their RMSD were displayed in Figure 5A. In the case of the doxycycline-loaded borneol-free formulation (F1), the water penetration occurred promptly within 5 ns as well as NMP, which mixed well with the water (Figure 4A,C). The RMSD curve of F1 with water reached equilibrium at about 35 ns and remained at 78 Å (Figure 5A). The absolute solvent exchange in which the drug was freely migrated suddenly occurred for F1 after contact with water due to a lack of matrix former in the formulation. For F2, water mixed well with NMP within 15 ns, and its RMSD became constant at 78 Å after 90 ns. Moreover, water in the system of F3 could be mixed well with NMP within 20 ns and F3 equilibrated at 78 Å after 125 ns. Whereas, the solvent exchange of F4 occurred within 100 ns and the RMSD of F4 with water increased slowly and attained the steady value of 55 Å in about 180 ns. It can be noticed that F4 with water used the highest simulation time to reach equilibrium, followed by F3, F2, and F1, implying that borneol and triacetin could slow the solvent exchange of the ISM formulation. Apparently, the rate of water infiltration was observed to decrease in correlation with the augmented quantity of triacetin. Moreover, borneol was employed as a matrix former in this study with the purpose of retard the solvent exchange mechanism. Regarding the previous experiments (Section 3.4, Section 3.5 and Section 3.6), the borneol-based formulations, clearly demonstrated a slower matrix formation, resulting in slower migration between water and doxycycline molecules and enabling controlled drug release. Additionally, triacetin, a hydrophobic substance, was used as an additive solvent to interrupt the solvent exchange, as can also be seen from the results mentioned above.

The RMSD of doxycycline in the simulation of F1–F4 models after contact with water was also explored to observe the topography change over time of doxycycline in each system, as shown in Figure 5B. The RMSD curve of F4 increased and fluctuated for the first 125 ns and became stable at 60 Å after 160 ns. Moreover, other formulations (F1–F3) with water had higher RMSD compared with F4 at the same time. Although the doxycycline in F1–F3 with water models reached equilibrium at a similar value of 69 Å, their duration times to became equilibrated were different; doxycycline in F1 with water took only 60 ns, followed by F2 and F3 (125 and 155 ns, respectively). A low and slowly increasing value of the RMSD reflects a tiny position change of molecules from the starting point compared to a high value of the RMSD. This indicated that the addition of borneol and triacetin in the formulation reflects the duration to reach equilibrium of doxycycline; hence, both of them have a direct effect on drug retardation. Additionally, the proportion of triacetin incorporated in ISM also affected the change in the position of the doxycycline molecular.

Typically, water molecules diffuse into the formulation after it contacts the crevicular fluid in the crevicular pocket. While the organic solvent from the formulation diffuses out into the environment, this process is known as a solvent exchange [3,50]. Therefore, the movement of NMP molecule as an organic solvent in this study was also important to observe to prove and understand the solvent exchange phenomenon. While the RMSD of the NMP conformation was analyzed and displayed in Figure 5C. As shown in the results, this curve of F4 reached equilibrium at 77 Å after 110 ns, while those of F2 and F3 became constant after 60 and 75 ns, respectively. The effects of borneol and triacetin not only retarded the drug movement but also affected the movement of the solvent in the formulation. By comparison of the RMSD of doxycycline and NMP conformation, the movement of the NMP molecule was faster than that of doxycycline, resulting in faster reaching the equilibrium of NMP (Figure 5C) because the molecular size of NMP is smaller than that of doxycycline. Although the RMSD of doxycycline and NMP conformation were constant within 200 ns, the RMSD of borneol conformation (Figure 5D) was slightly increased even after 200 ns. From the results of this experiment and the previous experiments, it can be concluded that this phenomenon was caused by the borneol gradually forming a matrix. Whereas some molecules of borneol still moved and were gradually induced by water for matrix transformation. Especially, the F4 formulation containing 25% triacetin, showed a slowed down exchange of solvent and water, resulting in the slowest phase transformation of borenol, as expressed in Figure 5D. Thus, the matrix transformation of the F4 formulation occurred gradually. Moreover, the diffusion constant of relevant molecules during the initial stages of matrix formation within nanoseconds was analyzed and displayed in Table 4 to prove the aforementioned hypothesis. It was noticed that the addition of borneol and triacetin minimized the diffusion constant of doxycycline, and affected the amount of water/NMP migrated (Figure 4A,C). Besides, the diffusion constant of NMP decreased upon the addition of borneol and/or triacetin. In comparison to F1, the impedetion of borneol clearly resulted in the decrease in the diffusion constant of both NMP and doxycycline in F2. The addition of triacetin to F3 also reduced the diffusion constant of both NMP and doxycycline. Although the addition of a hydrophobic solvent such as triacetin has the effect of ratarding solvent exchange, excessive addition of triacetin may result in incomplete or slow matrix formation because the solvent exchange process was interfered with by triacetin molecules. Eventually, the matrix was formed slowly (as discussed in Section 3.4, Section 3.5 and Section 3.6) and could not slow down the solvent exchange process. Therefore, the diffusion constant in NMP of F4 was slightly higher than that of F3. The addition of triacetin (F3) also reduced the diffusion constant of both NMP and doxycycline. Although the addition of a hydrophobic solvent such as triacetin retarded the solvent exchange, resulting in sustained drug release, excessive addition of triacetin may result in different ways. Incomplete matrix formation or too slow matrix formation could occur because the solvent exchange process was interfered with triacetin molecules. Eventually, the matrix was formed slowly (as discussed in Section 3.4, Section 3.5 and Section 3.6) and could not slow down the solvent exchange process. Therefore, the diffusion constant of NMP in F4 was slightly higher than that of F3. In addition, the diffusion constant of borneol decreased with the increasing amount of triacetin owing to the interfere of hydrophobic substance of triacetin. These results were consistent with MD and RMSD results (Figure 4 and Figure 5). In addition, the diffusion coefficient was also consistent with the matrix formation and drug release results, as shown in Section 3.4, Section 3.5 and Section 3.6.

**Figure 6:**
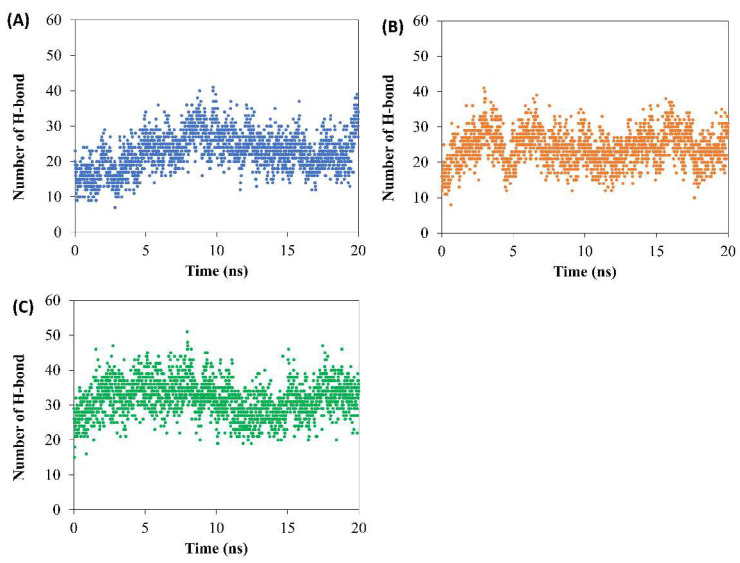
Change in number of hydrogen bonds between doxycycline and borneol molecule of (**A**) F2; (**B**) F3; and (**C**) F4 after formulations box contact with water.

During 200 ns of simulation time, the number of H-bonds between doxycycline and the borneol molecule of F2–F4 after contact with water was evaluated using VMD software, with the distance between the acceptor and donor atoms being less than 3.5 Å at less than 60° angle [33,34]. Ostensibly, the H-bond formation between borneol and doxycycline (Figure 6) in F4 had the highest trend value in the range of 20–40 NO., compared to those of the other two formulations that exhibited H-bonds in the range of 10–30 NO. Therefore, borneol in F4 had not yet self-aggregated, leaving more availability for H-bond formation with doxycycline compared to the other two formulations. This indirectly supported that the borneol matrix of F2 and F3 were better formed than that of F4. These simulations confirmed the authors’ claim on the aforementioned matrix formation from both macro/micro level studies [7,11,12,25].

According to the simulation results (MD), the borneol with or without triacetin retard both doxycycline and NMP migration. The addition of borneol and/or triacetin showed that matrices self-assembled of F2 and F3 were faster than that of F4 as shown in the following results: RMSD of NMP (Figure 5C), RMSD of borneol (Figure 5D), and the decrease of diffusion constant of borneol (Table 4). In addition, the change in the number of hydrogen bonds between doxycycline and borneol molecule of F2–F4 indicated that the formation of the borneol matrix of F2 and F3 was better than that of F4. The results from molecular dynamic modelling, self-formation ability, interfacial network formation and in vitro release of ISMs supported each other. As a result, F3, a borneol-based formula containing 5% triacetin, could be a promising in situ forming system for drug delivery system due to its outstanding self-assembled time and ability to prolong drug release compared to others.

## 4. Conclusions

These findings were consistent with the experiments conducted at the macroscopic level. The increase in triacetin resulted in fewer topographical changes occurring during self-assembly. Therefore, computer simulations may be useful in considering the possibilities of various experiments. Hence, the process of in situ formation at both macro and micro levels was explained. Firstly, when the water and system interacted, a mixed water/solvent region was formed due to the penetration of water into the system and solvent into the water phase. Secondly, the initial nucleation of matrix forming agents such as borneol was formed in the high-water-ratio area, where the presence of hydrophobic liquids such as triacetin affected the water ratio and reduced water diffusion. Subsequently, this resulted in different nucleation origins and matrix growth rates. At the molecular level, drug and NMP movement depended on the ratio of triacetin, both tried to escape from the region of the matrix. Borneol mass growth continued, leading to complex/non-complex interior structures of matrices depending on the triacetin ratio and nucleation origin number. The drug laid itself down with the borneol matrix structure, resulting in burst release for the following reasons: burst release from the drug that laid on the external site of the matrix, aggressive movement of solvent that carried the drug, high porous and less tortuous structure of the matrix. The molecular-level simulation could be used to verify the phenomenon of matrix formation and reveal the in situ formation mechanism at the initial time. The results of the experiments all supported each other. It was also found that formulation F3 is the most effective, considering the following factors: the ability to control drug release, the ability to sustain drug release, viscosity, the ability to form a matrix, and the time of the in situ forming process. The addition of either borneol or triacetin delayed the drug’s release. However, too much triacetin, as in F4, has the opposite effect. Namely, instead of slowing the drug’s release, it accelerated the release. Because it causes the matrix to form more slowly, which the drug is easily diffused outward during incomplete of solid structure formation. As demonstrated in Section 3.4 and Section 3.5, when the matrix forms slowly, it may be unable to control drug release (as shown in Section 3.6). Furthermore, the MD simulation results in the first 200 nanoseconds produce the same results. As a result, the best formulation in this study is 5% triacetin with borneol-based ISM.

## Figures and Tables

**Table 1:** Compositions of in situ forming matrix systems.

Formulation	Amount (% *w*/*w*)			
	Doxycycline Hyclate	Borneol	Triacetin	NMP
F1	5	-	-	95
F2	5	40	-	55
F3	5	40	5	50
F4	5	40	25	30

**Table 2:** Box design details of all models used in the simulation processes.

(**A**) Box design of formulation before contact with water box				
Details	F1	F2	F3	F4
Amount of Dox molecules	80	80	80	80
Amount of Bor molecules	0	1840	1840	1840
Amount of Tri molecules	0	0	160	800
Amount of NMP molecules	6800	4000	3600	2160
Mole ratio of Dox:Bor:Tri:NMP	1:0:0:85	1:23:0:50	1:23:2:45	1:23:10:27
Total amount of molecules in system	6880	5920	5680	4880
Total amount of atom in system	113,280	121,840	120,080	115,600
Box size (x, y, z)	112, 104, 120	140, 116, 132	140, 132, 140	140, 116, 124
(**B**) Box design of formulation after contact with water box				
Details	F1	F2	F3	F4
Amount of Dox molecules	80	80	80	80
Amount of Bor molecules	0	1840	1840	1840
Amount of Tri molecules	0	0	160	800
Amount of NMP molecules	6800	4000	3600	2160
Amount of Wat molecules	7920	7920	7920	7920
Mole ratio of Dox:Bor:Tri:NMP:Wat	1:0:0:85:99	1:23:0:50:99	1:23:2:45:99	1:23:10:27:99
Total amount of molecule in system	14,800	13,840	13,600	12,800
Total amount of molecule in system	137,040	145,600	143,840	139,360
Box size (x, y, z)	112, 104, 147	140, 116, 159	140, 132, 167	140, 116, 151

Remark: Dox = doxycycline, Bor = borneol, Tri = triacetin and Wat = water.

**Table 3:** Physical properties of triacetin, NMP, and borneol-based ISMs in liquid state (*n* = 3).

Formulation	Density (g/cm^3^)	Viscosity (cPs)	Surface Tension (mN/m)
Triacetin	1.1472 ± 0.0002	25.53 ± 0.52	38.87 ± 0.55
NMP	1.0283 ± 0.0004	1.88 ± 0.03	39.62 ± 0.02
F1	1.0425 ± 0.0009	2.59 ± 0.16	40.33 ± 0.42
F2	1.0204 ± 0.0014 ^a,b^	5.38 ± 0.09 ^c,d^	35.18 ± 0.16
F3	1.0268 ± 0.0017 ^a^	6.16 ± 0.07 ^c^	34.61 ± 0.22
F4	1.0435 ± 0.0013 ^b^	9.67 ± 0.04 ^d^	33.14 ± 0.09

The superscripts ^a^, ^b^, ^c^, and ^d^ in the columns represent a significant difference (*p* < 0.05).

**Table 4:** Diffusion constant of each substance in formulations box contact with water.

Substance	Diffusion Constant (m^2^/s)			
	F1	F2	F3	F4
Doxycycline	0.52	0.32	0.24	0.18
Borneol	-	0.60	0.51	0.41
Triacetin	-	-	0.63	0.61
NMP	2.24	1.72	1.40	1.43

## Data Availability

The data presented in this study are available on the request from the corresponding author.
